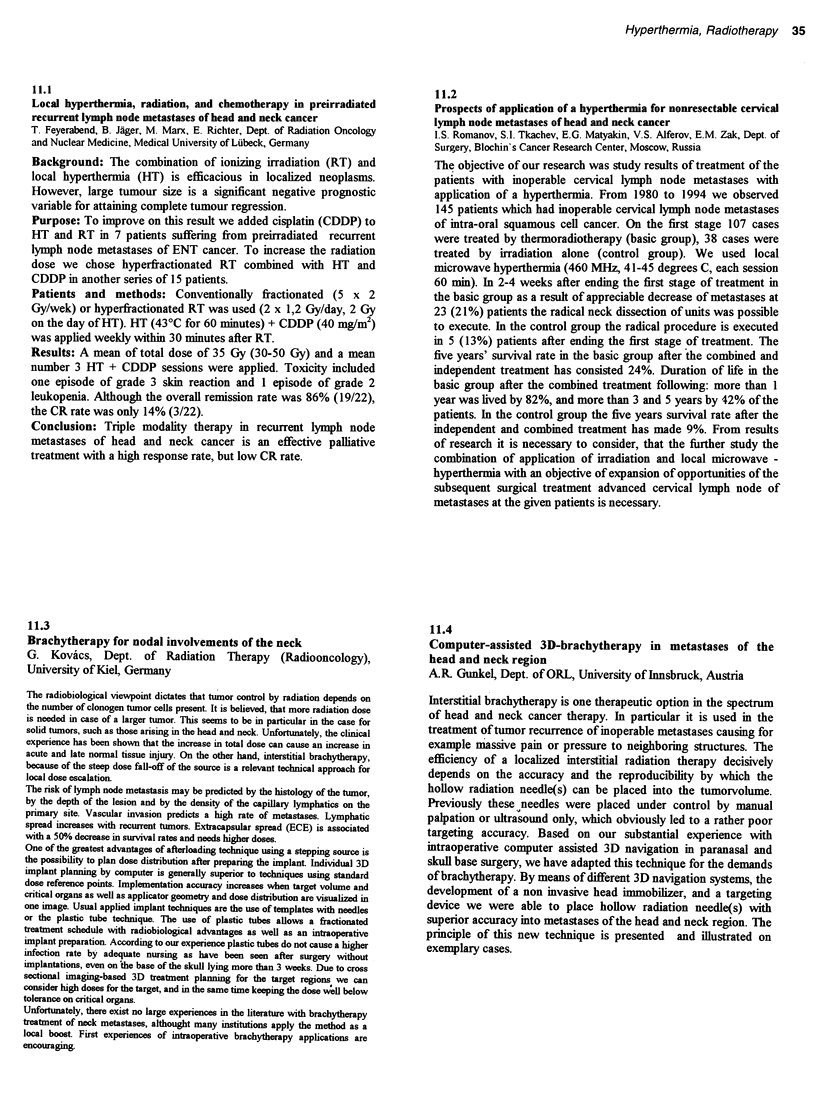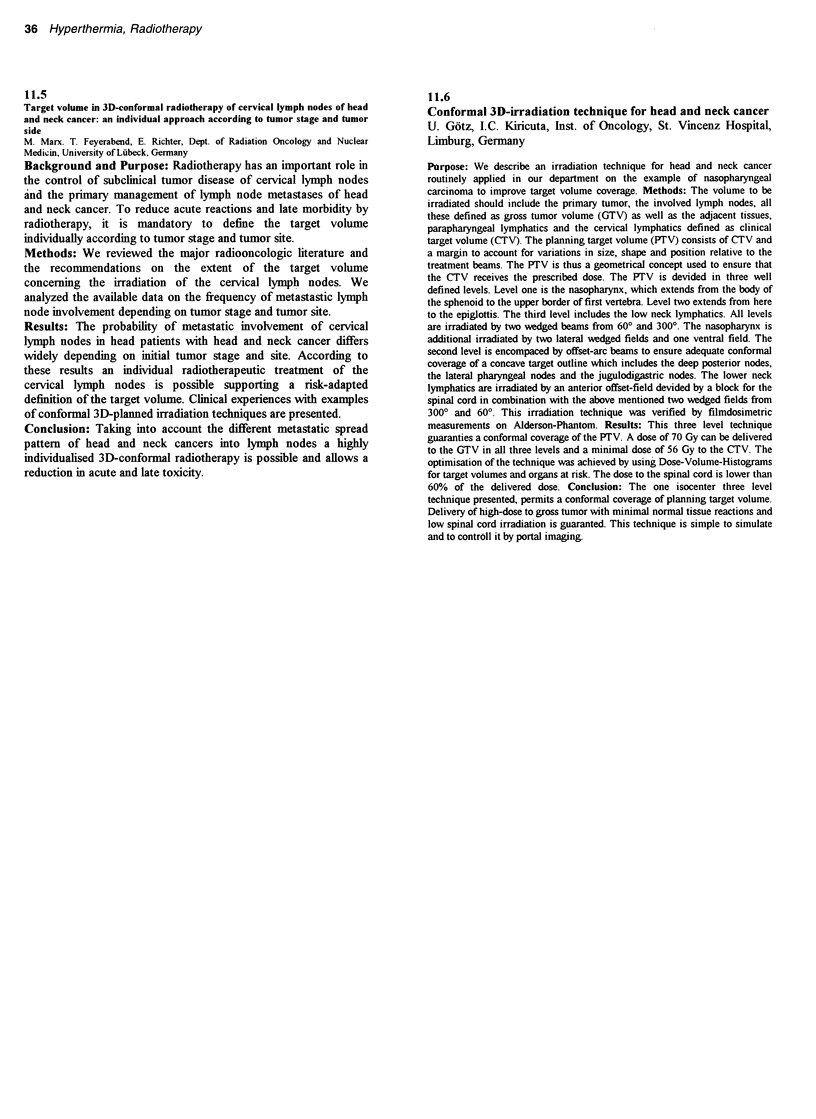# Hyperthermia, Radiotherapy

**Published:** 1998

**Authors:** 


**Local hyperthermia, radiation, and chemotherapy in preirradiated recurrent lymph node metastases of head and neck cancer**

T. Feyerabend, B. Jäger, M. Marx, E. Richter, Dept. of Radiation Oncology and Nuclear Medicine, Medical University of Lübeck, Germany

**Background:** The combination of ionizing irradiation (RT) and local hyperthermia (HT) is efficacious in localized neoplasms. However, large tumour size is a significant negative prognostic variable for attaining complete tumour regression.

**Purpose:** To improve on this result we added cisplatin (CDDP) to HT and RT in 7 patients suffering from preirradiated recurrent lymph node metastases of ENT cancer. To increase the radiation dose we chose hyperfractionated RT combined with HT and CDDP in another series of 15 patients.

**Patients and methods:** Conventionally fractionated (5 x 2 Gy/wek) or hyperfractionated RT was used (2 x 1,2 Gy/day, 2 Gy on the day of HT). HT (43°C for 60 minutes) + CDDP (40 mg/m$^2$) was applied weekly within 30 minutes after RT.

**Results:** A mean of total dose of 35 Gy (30-50 Gy) and a mean number 3 HT + CDDP sessions were applied. Toxicity included one episode of grade 3 skin reaction and 1 episode of grade 2 leukopenia. Although the overall remission rate was 86% (19/22), the CR rate was only 14% (3/22).

**Conclusion:** Triple modality therapy in recurrent lymph node metastases of head and neck cancer is an effective palliative treatment with a high response rate, but low CR rate.

**11.2**

**Prospects of application of a hyperthermia for nonresectable cervical lymph node metastases of head and neck cancer**

I.S. Romanov, S.I. Tkachev, E.G. Matyakin, V.S. Alferov, E.M. Zak, Dept. of Surgery, Blochin's Cancer Research Center, Moscow, Russia

The objective of our research was study results of treatment of the patients with inoperable cervical lymph node metastases with application of a hyperthermia. From 1980 to 1994 we observed 145 patients which had inoperable cervical lymph node metastases of intra-oral squamous cell cancer. On the first stage 107 cases were treated by thermoradiotherapy (basic group), 38 cases were treated by irradiation alone (control group). We used local microwave hyperthermia (460 MHz, 41-45 degrees C, each session 60 min). In 2-4 weeks after ending the first stage of treatment in the basic group as a result of appreciable decrease of metastases at 23 (21%) patients the radical neck dissection of units was possible to execute. In the control group the radical procedure is executed in 5 (13%) patients after ending the first stage of treatment. The five years' survival rate in the basic group after the combined and independent treatment has consisted 24%. Duration of life in the basic group after the combined treatment following: more than 1 year was lived by 82%, and more than 3 and 5 years by 42% of the patients. In the control group the five years survival rate after the independent and combined treatment has made 9%. From results of research it is necessary to consider, that the further study the combination of application of irradiation and local microwave - hyperthermia with an objective of expansion of opportunities of the subsequent surgical treatment advanced cervical lymph node of metastases at the given patients is necessary.

**11.3**

**Brachytherapy for nodal involvements of the neck**

G. Kovács, Dept. of Radiation Therapy (Radiooncology), University of Kiel, Germany

The radiobiological viewpoint dictates that tumor control by radiation depends on the number of clonogen tumor cells present. It is believed, that more radiation dose is needed in case of a larger tumor. This seems to be in particular in the case for solid tumors, such as those arising in the head and neck. Unfortunately, the clinical experience has been shown that the increase in total dose can cause an increase in acute and late normal tissue injury. On the other hand, interstitial brachytherapy, because of the steep dose fall-off of the source is a relevant technical approach for local dose escalation.

The risk of lymph node metastasis may be predicted by the histology of the tumor, by the depth of the lesion and by the density of the capillary lymphatics on the primary site. Vascular invasion predicts a high rate of metastases. Lymphatic spread increases with recurrent tumors. Extracapsular spread (ECE) is associated with a 50% decrease in survival rates and needs higher doses.

One of the greatest advantages of afterloading technique using a stepping source is the possibility to plan dose distribution after preparing the implant. Individual 3D implant planning by computer is generally superior to techniques using standard dose reference points. Implementation accuracy increases when target volume and critical organs as well as applicator geometry and dose distribution are visualized in one image. Usual applied implant techniques are the use of templates with needles or the plastic tube technique. The use of plastic tubes allows a fractionated treatment schedule with radiobiological advantages as well as an intraoperative implant preparation. According to our experience plastic tubes do not cause a higher infection rate by adequate nursing as have been seen after surgery without implantations, even on the base of the skull lying more than 3 weeks. Due to cross sectional imaging-based 3D treatment planning for the target regions we can consider high doses for the target, and in the same time keeping the dose well below tolerance on critical organs.

Unfortunately, there exist no large experiences in the literature with brachytherapy treatment of neck metastases, althought many institutions apply the method as a local boost. First experiences of intraoperative brachytherapy applications are encouraging.

**11.4**

**Computer-assisted 3D-brachytherapy in metastases of the head and neck region**

A.R. Gunkel, Dept. of ORL, University of Innsbruck, Austria

Interstitial brachytherapy is one therapeutic option in the spectrum of head and neck cancer therapy. In particular it is used in the treatment of tumor recurrence of inoperable metastases causing for example massive pain or pressure to neighboring structures. The efficiency of a localized interstitial radiation therapy decisively depends on the accuracy and the reproducibility by which the hollow radiation needle(s) can be placed into the tumorvolume. Previously these needles were placed under control by manual palpation or ultrasound only, which obviously led to a rather poor targeting accuracy. Based on our substantial experience with intraoperative computer assisted 3D navigation in paranasal and skull base surgery, we have adapted this technique for the demands of brachytherapy. By means of different 3D navigation systems, the development of a non invasive head immobilizer, and a targeting device we were able to place hollow radiation needle(s) with superior accuracy into metastases of the head and neck region. The principle of this new technique is presented and illustrated on exemplary cases.

### 11.5

**Target volume in 3D-conformal radiotherapy of cervical lymph nodes of head and neck cancer: an individual approach according to tumor stage and tumor side**

M. Marx. T. Feyerabend, E. Richter, Dept. of Radiation Oncology and Nuclear Medicin, University of Lübeck, Germany

**Background and Purpose:** Radiotherapy has an important role in the control of subclinical tumor disease of cervical lymph nodes and the primary management of lymph node metastases of head and neck cancer. To reduce acute reactions and late morbidity by radiotherapy, it is mandatory to define the target volume individually according to tumor stage and tumor site.

**Methods:** We reviewed the major radiooncologic literature and the recommendations on the extent of the target volume concerning the irradiation of the cervical lymph nodes. We analyzed the available data on the frequency of metastastic lymph node involvement depending on tumor stage and tumor site.

**Results:** The probability of metastatic involvement of cervical lymph nodes in head patients with head and neck cancer differs widely depending on initial tumor stage and site. According to these results an individual radiotherapeutic treatment of the cervical lymph nodes is possible supporting a risk-adapted definition of the target volume. Clinical experiences with examples of conformal 3D-planned irradiation techniques are presented.

**Conclusion:** Taking into account the different metastatic spread pattern of head and neck cancers into lymph nodes a highly individualised 3D-conformal radiotherapy is possible and allows a reduction in acute and late toxicity.

### 11.6

**Conformal 3D-irradiation technique for head and neck cancer**

U. Götz, I.C. Kiricuta, Inst. of Oncology, St. Vincenz Hospital, Limburg, Germany

**Purpose:** We describe an irradiation technique for head and neck cancer routinely applied in our department on the example of nasopharyngeal carcinoma to improve target volume coverage. **Methods:** The volume to be irradiated should include the primary tumor, the involved lymph nodes, all these defined as gross tumor volume (GTV) as well as the adjacent tissues, parapharyngeal lymphatics and the cervical lymphatics defined as clinical target volume (CTV). The planning target volume (PTV) consists of CTV and a margin to account for variations in size, shape and position relative to the treatment beams. The PTV is thus a geometrical concept used to ensure that the CTV receives the prescribed dose. The PTV is devided in three well defined levels. Level one is the nasopharynx, which extends from the body of the sphenoid to the upper border of first vertebra. Level two extends from here to the epiglottis. The third level includes the low neck lymphatics. All levels are irradiated by two wedged beams from 60° and 300°. The nasopharynx is additional irradiated by two lateral wedged fields and one ventral field. The second level is encompaced by offset-arc beams to ensure adequate conformal coverage of a concave target outline which includes the deep posterior nodes, the lateral pharyngeal nodes and the jugulodigastric nodes. The lower neck lymphatics are irradiated by an anterior offset-field devided by a block for the spinal cord in combination with the above mentioned two wedged fields from 300° and 60°. This irradiation technique was verified by filmdosimetric measurements on Alderson-Phantom. **Results:** This three level technique guaranties a conformal coverage of the PTV. A dose of 70 Gy can be delivered to the GTV in all three levels and a minimal dose of 56 Gy to the CTV. The optimisation of the technique was achieved by using Dose-Volume-Histograms for target volumes and organs at risk. The dose to the spinal cord is lower than 60% of the delivered dose. **Conclusion:** The one isocenter three level technique presented, permits a conformal coverage of planning target volume. Delivery of high-dose to gross tumor with minimal normal tissue reactions and low spinal cord irradiation is guaranted. This technique is simple to simulate and to controll it by portal imaging.